# Synthesis, Characterization and Application of Iron(II) Doped Copper Ferrites (Cu^II^_(x)_Fe^II^_(1-x)_Fe^III^_2_O_4_) as Novel Heterogeneous Photo-Fenton Catalysts

**DOI:** 10.3390/nano10050921

**Published:** 2020-05-09

**Authors:** Asfandyar Khan, Zsolt Valicsek, Ottó Horváth

**Affiliations:** 1 Department of General and Inorganic Chemistry, Faculty of Engineering, University of Pannonia, Egyetem utca 10, H-8200 Veszprém, Hungary; asfandyarkhan100@gmail.com (A.K.); valicsek@almos.uni-pannon.hu (Z.V.); 2 Department of Textile Processing, National Textile University, Faisalabad 37610, Pakistan

**Keywords:** heterogeneous photo-Fenton type system, iron(II) doped copper ferrite, photocatalytic-degradation, methylene blue (MB)

## Abstract

The heterogeneous photo-Fenton type system has huge fame in the field of wastewater treatment due to its reusability and appreciable photoactivity within a wide pH range. This research investigates the synthesis and characterization of iron(II) doped copper ferrite (Cu^II^_(x)_Fe^II^_(1-x)_Fe^III^_2_O_4_ nanoparticles (NPs) and their photocatalytic applications for the degradation of methylene blue (MB) as a model dye. The NPs were prepared via simple co-precipitation technique and calcination. The NPs were characterized by using Raman spectroscopy, X-ray diffractometry (XRD), scanning electron microscopy (SEM), and diffuse reflectance spectroscopy (DRS). SEM reveals the structural change from the spherical-like particles into needle-like fine particles as the consequence of the increasing ratio of copper(II) in the ferrites, accompanied by the decrease of the optical band-gap energies from 2.02 to 1.25 eV. The three major determinants of heterogeneous photo-Fenton system, namely NPs concentration, hydrogen peroxide concentration and pH, on the photocatalytic degradation of MB were studied. The reusability of NPs was found to be continuously increasing during 4 cycles. It was concluded that iron(II) doped copper ferrites, due to their favorable band-gap energies and peculiar structures, exhibit a strong potential for photocatalytic-degradation of dyes, for example, MB.

## 1. Introduction

The conventional (physical, biological, and chemical) wastewater (WW) treatment methods are costly, inefficient, complex, and produce secondary pollution [1,2,3]. Recently, advanced oxidation processes (AOPs) have been investigated due to the use of hydroxyl radicals (HO^●^) formation, which exhibits strong oxidation capacity (E_0_ = 2.76 V) [4], for example, homogeneous and heterogeneous Fenton [5,6], photo-Fenton, ozonation [7], hydrogen peroxide with ozone [8], hydrogen peroxide with UV [9], titanium dioxide/UV [10], zinc oxide/UV [11], and so forth.

The most frequently practiced method is the homogeneous Fenton type process, where a catalyst (Fe^2+^) and oxidant H_2_O_2_ is added to the WW for treatment [12,13,14]. The simplicity, efficient performance and the non-toxicity of Fenton method are its major advantages [15,16]; hence, it is fruitfully applied in the textile [17] and pharmaceutical [18] industry, olive-oil mills [19], production of insecticides and pesticides [20], pulp mills [21], and phenolic WW [22]. Besides the wide applications and advantages of homogeneous Fenton type process, it also has some disadvantages, for example, restricted operating pH, heavy sludge formation, huge operating and maintenance cost, and limited recyclability of the catalyst [23]. Research attention has been diverted towards the synthesis of heterogeneous catalysts which can work in a wider pH range, at low operating cost, with better performance, and easy to separate after usage. Heterogeneous, ferrite-type catalysts can be applied in photo-Fenton, electro-Fenton, and photo-electro (PE) Fenton processes, and they can be reused many times [4,24,25,26,27,28,29].

The literature has reported two types of heterogeneous Fenton catalysts, that is, (1) iron catalyst supported on other materials (Fe absorbed on Nafion membranes, and ZSM5 zeolite) and (2) iron oxides (Fe_2_O_3_, Fe_2_V_4_O_13_, and some V, Cu, Ce, Nb, and Si doped iron oxides) [30,31,32,33,34]. For example, cobalt ferrites doped with nickel [35], zinc ferrites [36], aluminum doped zinc ferrites [37] and copper ferrites [38] were successfully used for the photocatalytic degradation of several dyes and other pollutants (e.g., nitroaromatic compounds). Few among them have antibacterial activity, too [39], [40]. According to these observations, doped, composite-type ferrites seem to exhibit better catalytic potentials than undoped ones. Nevertheless, no study has been reported about the iron(II) doped copper ferrites; therefore, the main aim of our research was the synthesis, characterization, and application of iron(II) doped copper ferrites as novel catalysts for the degradation of Methylene Blue (MB) as a model compound (Figure S1 in the Supplementary Materials (SM)).

## 2. Materials and Methods

### 2.1. Materials

Ammonium iron(II) sulfate hexahydrate, ferric chloride hexahydrate, copper(II) sulfate pentahydrate, Methlyene Blue (systematic name: methylthioninium chloride) and sodium hydroxide were used in analytical grade without further purification. Hydrogen peroxide (30% w/w) was applied as a Fenton reagent. The pH was adjusted by the addition of sodium hydroxide or hydrochloric acid. All reagents were purchased from Sigma-Aldrich (Budapest, Hungary). Synthesized NPs were purified by ethanol (absolute) and double distilled water.

### 2.2. Synthesis of NPs

The NPs were synthesized with change of Cu^2+^ and Fe^2+^ in the composition given as Cu^II^_(x)_Fe^II^_(1-x)_Fe^III^_2_O_4_ (where x = 0.0, 0.2, 0.4, 0.6, 0.8, 1.0). NPs were synthesized by using a simple co-precipitation method. In this method, which was suggested by Singh et al. [35], solution I was prepared by adding Fe(NH_4_)_2_(SO_4_)_2_∙6H_2_O, FeCl_3_∙6H_2_O, and CuSO_4_ salts to 20 mL distilled water in stoichiometric amounts as shown in Table 1 and sonicated for 30 min at room temperature, while solution II was 5 M NaOH (20 mL), which acted as precipitating agent. Solutions I and II were mixed together dropwise and were stirred continuously by using magnetic stirrer for 60 min. Table 1 indicates the theoretical stoichiometric compositions of the catalysts prepared.

The dark precipitates obtained were purified by using ethanol and double distilled water under a centrifugal filtration technique. The applied centrifugal filtration was basically a centrifugation and re-dispersion process repeated twice with ethanol and twice with double distilled water. The purified solid hydroxides, as precursors, were dried in an oven at 110 °C for 60 min. Then the dried NPs were calcined at 400 °C for 4 h. The simple metal oxides (CuO, FeO, and Fe_2_O_3_) were prepared in a similar way.

Although the very low values of the solubility product constants regarding the corresponding hydroxides (see Text S1 in the SM [41]) suggested that the total amounts of the metal ions weighed in were precipitated in the NaOH excess during the syntheses, the Cu/Fe ratios in the final products (after calcination) were determined by ICP measurements. As shown in Table S1, the deviations of the experimental values from the theoretical ones are within 5%.

The powdered catalysts were characterized and used for photocatalytic degradation of MB under visible-light irradiation.

### 2.3. Characterization of NPs

For determination of the experimental Cu/Fe ratios of the catalysts prepared, the concentration of metal ions were analyzed by using inductively coupled optical emission spectrometry (ICP-OES), with the application of Perkin Elmer Optima 2000 DV equipment (PerkinElmer Inc., Waltham, MA, USA). Standard solutions were prepared from 1 g/L of each metal (Merck standard solutions), and freshly diluted before use. The linear regression method was used to calculate the calibration curves. The monitoring wavelengths were 327.393 and 238.204 nm for Cu and Fe, respectively. The X-ray diffraction (XRD) patterns of the Cu^II^_(x)_Fe^II^_(1-x)_Fe^III^_2_O_4_ NPs were measured by using a Philips PW 3710 type powder diffractometer (Philips Analytical B.V., Almelo, Netherlands) with a graphite diffracted-beam monochromator and CuK_α_ radiation (λ = 0.1541 nm) generated at 50 kV and 40 mA. The samples were measured in a continuous scan mode with 0.02°/sec scanning speed. Data collections and evaluations were carried out with an X’Pert Data Collector (v.: 2.0e) and an X’Pert High Score Plus software. (v.: 2.2e (2.2.5), PANanalytical B.V., Almelo, Netherlands) The specific surface areas of the catalysts were determined by the Brunauer-Emmett-Teller (BET) method from N_2_ adsorption/desorption isotherms, using a Micromeritics ASAP 2000 type instrument. (Micromeritics Instrument Corporation, Norcross, GA, USA) For each measurement, 1 g sample was previously outgassed in vacuum at 160 °C. For the Raman spectroscopic measurements, a Bruker RFS 100/S FT—Raman spectrometer (Bruker Corporation, Billerica, MA, USA) equipped with Nd:YAG laser (1064 nm, operated at 150 mW) and a liquid N_2_ cooled Ge-diode detector was used. The spectra of the powdered samples were recorded by the co-addition of 512 scans with a resolution of 2 cm^−1^. Thermo Scientific™ scanning electron microscope (SEM) (Thermo Fisher Scientific Inc., Waltham, MA, USA)), model APREO S with accumulated voltage of 20 kV, beam current in the range 0.80–1.60 mA and low vacuum secondary electron detector was used to study the surface morphology. Energy dispersive X-ray (EDX) spectral analysis was performed for uncoated samples by an EDAX AMETEK (Mahwah, NJ, USA) equipment with an octane detector using TEAM™ software (v.: 4.5, EDAX AMETEK Inc., Mahwah, NJ, USA). A Zetasizer NanoZS (Malvern Instruments Ltd, Malvern, Worcestershire, UK.) dynamic light scattering instrument was applied for the measurement of particle size distribution (PSD). Diffuse reflectance spectroscopy (DRS) was used to determine the band-gap energy of the prepared NPs. The scattering spectra were recorded by a Perkin Elmer LS50 B spectrofluorometer (PerkinElmer Inc., Waltham, USA) in solid phase at a wavelength range of 250–600 nm. Barium sulfate was the reference (I_0_) to measure the reflectance (R), then by the use of the Kubelka-Munk function [42], the values of which must be presented depending on the excitation energy (in eV = electron volt), the cross-section point of the extrapolated linear portion of the curve on to *X*-axis (see Figure S2) will give the band-gap energy for the powder sample Equations (1)and (2):

(1) $$R=\frac{I}{I_{0}}$$

(2) $$f\left(R\right)=\frac{{\left(1-R\right)}^{2}}{2R}$$

### 2.4. Assessment of Photocatalytic Activity

**Figure 1:**
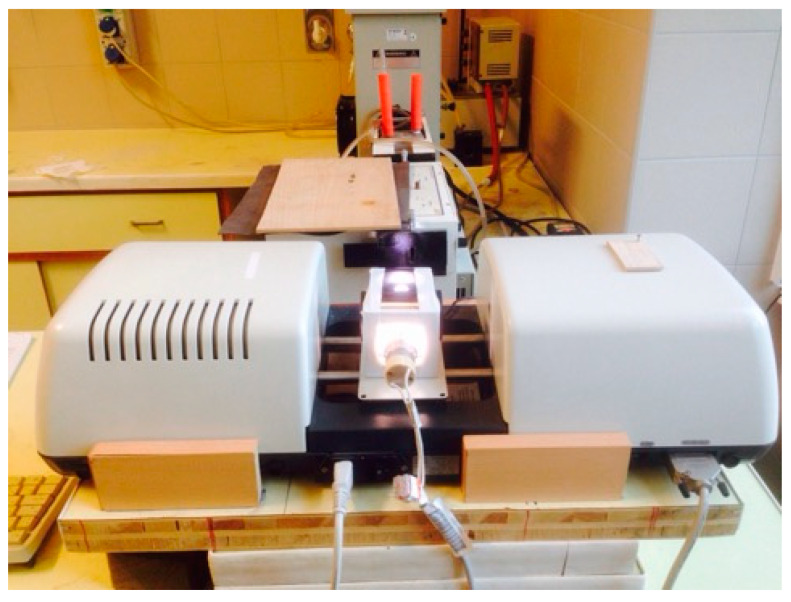
Assembly for photocatalytic experiments.

The photocatalytic degradation of MB was carried out under visible-light irradiation by the use of an Optonica SP1275 LED lamp (GU10, 7 W, 400 Lm, 6000 K, Optonica LED, Sofia, Bulgaria) at room temperature in a 1-cm pathlength quartz cuvette fitted directly inside a S600 diode-array spectrophotometer as shown in Figure 1. The volume of the reaction mixture in the cuvette irradiated was 3 cm^3^ in all cases. The concentration of MB (1.5 × 10^−5^ mol/L) and the duration (140 min) of the photocatalytic experiments were constant throughout the research. First of all, the potential self-degradation of MB with and without light (in dark) was checked. The previous observations published in the literature [43,44] also confirmed that MB was stable in dark, but photosensitive to the visible light. We determined the reaction rate of this photo-induced self-degradation of MB compared to that of the photoreaction, in which we used H_2_O_2_ as oxidant reagent in the concentration (0.01 M) suggested in similar works published in the literature [35]. After that the effect of the presence of nanoparticles (e.g., NP-3) as heterogeneous photocatalysts was checked. Self-degradation of MB will be ignored in the subsequent studies. The mixture of MB and catalyst was continuously stirred in a dark room for 30 min in order to achieve adsorption/desorption equilibrium. Subsequently, the quartz cuvette was set in the sample holder of the spectrophotometer and irradiated by visible light. The solution inside the cuvette was continuously stirred by using magnetic stirrer. The reaction was initiated by the addition of commercial H_2_O_2_ and opening the window for the light source. The absorption spectra were measured continuously from the start until the end of the process. The effects of NPs concentration, H_2_O_2_ concentration, and pH on the MB degradation were investigated. Additionally, the reusability of NPs in the photocatalytic system was also studied.

The degradation efficiency was calculated by using the Beer Lambert law (Equation (3)),

(3) $$A_{\lambda ,t}=\varepsilon_{\lambda }c_{t}\ell ,$$

where A is the absorbance as the function of wavelength (λ, in unit “nm”) and time (t, in unit “s”), ε is the molar absorbance of dye (M^−1^ cm^−1^) as the function of wavelength, c is the concentration of dye (M) in the solution (as the function of time during the photolysis) and ℓ is the path length of the cuvette (cm).

The reaction rate (dc/dt) was calculated from the change of absorbances, dA/dt (1/s), which is the slope (m) of the degradation absorption curve (A vs. t, Equation (4)).

(4) $$\frac{\mathrm{dc}}{\mathrm{dt}}=\frac{\frac{\mathrm{dA}}{\mathrm{dt}}}{\varepsilon \;\ell }$$

On the basis of the spectral changes (see later in Section 3.2.), it can be declared that the intermediates and end-products of the photodegradation of MB have no significant absorption bands in the visible range; hence, the reaction rate of the MB degradation could simply be determined from the decrease of absorbances at the wavelength of its main absorption peak (665 nm). Moreover, the addition of NPs to the reaction mixture caused changes in the baselines of the measured spectra as the consequence of scattering. This problem was eliminated by the use of linear baseline corrections during the determination of the reaction rate.

The possible leaching of metal ions during the irradiation was checked for the NP-3 catalyst (after its use under optimum conditions) by both ICP measurements and spectrophotometry. In the latter case, suitable ligands (such as SCN^-^ for Fe^3+^ or phenanthroline for Fe^2+^ and Cu^2+^) were applied, by which the detection limits were 4.8 × 10^−7^ M, 9.0 × 10^−7^ M, and 3.3 × 10^−7^ M, taken 0.01 as minimum detectable absorbance. The total release of metal ions observed in the solution phase after the removal of the dispersed catalyst was much below 1%. This observation was confirmed by ICP measurements, according to which the concentrations of the dissolved copper and iron were 175 ± 9.8 µg/L and 672.5 ± 28.4 µg/L, respectively. These values correspond to 0.404 ± 0.023% and 0.272 ± 0.011%, respectively, taking 400 mg/L for the dispersed catalyst into account.

### 2.5. Assessment of Reusability

The reusability is one of the major points to be considered in the heterogeneous Fenton system. A five-step experiment was carried out in the quest of analyzing the reusability of NP-3 at appropriate conditions. After the complete degradation of MB in the cuvette, the system was allowed to stand for a night in dark for the total decay of hydrogen peroxide in the solution. Next day, the same amount of MB and H_2_O_2_ was added to the cuvette and the reaction was started again for a similar time interval. The procedure was repeated five times and the apparent kinetic constant was calculated from the reaction rate.

## 3. Results

### 3.1. Characterization of Cu^II^_(x)_Fe^II^_(1-x)_Fe^III^_2_O_4_ NPs

The results regarding the particle size distribution (as shown in Figure S3) clearly indicated that our catalysts were of submicrometer size, predominantly in the 70–200-nm range, which was favorable for the preparation of homogeneous aqueous dispersions.

**Figure 2:**
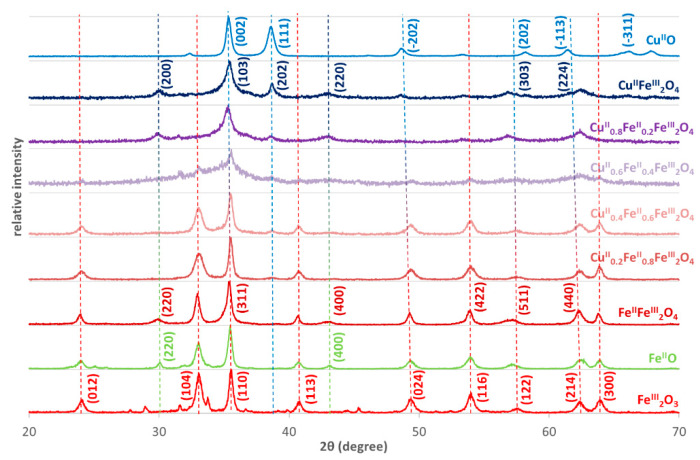
X-ray diffraction (XRD) diffractograms of iron(II) doped copper ferrites compared to those of the simple oxides of the given metal ions. The characteristic Miller indices indicated for the compounds the standards of which were earlier studied by XRD are taken from the International Centre for Diffraction Data.

Copper ferrites exhibit inverse spinel (instead of spinel) structure: metal ions with +2 charge (Fe^2+^ or Cu^2+^) are in octahedral position, while the half of the Fe^3+^ ions are in tetrahedral one [45]. This structure does not change during the substitution of Cu(II) ions to Fe(II) in the iron(II) doped copper ferrites. This is confirmed by the very slight change in the main peak at about 35 deg (2θ) in the XRD diffractograms (Figure 2)—35.6 deg in Fe_2_O_3_ (hematite) for the the crystal plane with (110) Miller indices, 35.4 deg in Fe_3_O_4_ (magnetite, x = 0 NP-1) for (311) crystal plane, 35.9 in CuFe_2_O_4_ (copper ferrite, x = 1 NP-6) for (211) crystal plane, 35.5 deg in CuO (tenorite) for (002) crystal plane.The positions of few common peaks change slightly stronger (shift) at about 58 and 63 deg, owing to the small difference in the size of the metal ions—iron(II) ions have 77 pm ionic radius in tetrahedral, and 92 pm in octahedral coordination geometry, while copper(II) 71, and 87 pm, respectively [46]. Already in the diffractogram of magnetite (Fe_3_O_4_, x = 0, NP-1), new peaks appeared compared to that of hematite (Fe_2_O_3_), as the consequence of the presence of metal ions with +2 oxidation state in the ferrite structure: at 30 and 43 deg. These peaks can be observed and assigned in the diffractograms of all nanoparticles, mainly in those of NP-1, NP-5 and NP-6, but their intensities were low in NPs 2-4, however, totally missed from Fe_2_O_3_ and CuO. This phenomenon may confirm the significant structural changes in the composites compared to the simple metal oxides. On the basis of the XRD evaluation, our FeO sample contained not only wüstite fraction, rather also maghemite, as the consequence of the potential partial oxidation of Fe^2+^ ions to Fe^3+^ during the calcination.

**Figure 3:**
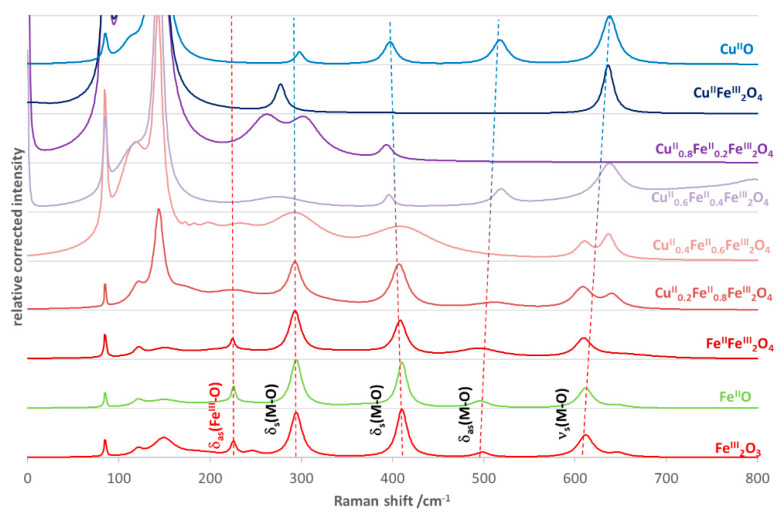
Raman spectra of iron(II) doped copper ferrites compared to those of the simple oxides of the given metal ions.

There are several peaks in Figure 3 (at 24, 33, 41, 49, 64 deg), which belong to hematite without the possible assignment to the magnetite. This means that a partially separated hematite fraction is in NPs 1-4 with a decreasing ratio, together with the increase of the Cu^2+^ content. However, tenorite did not compose a distinct fraction in a significant measure, not even in NP-6.

Also the Raman spectra of NPs confirm the inverse spinel structure (Figure 3). The vibrations under 600 cm^−1^ correspond to the M–O bonds at the octahedral sphere [47]. Only one band belongs to the metal ions with tetrahedral coordination sphere—the symmetric stretching at 610 cm^−1^ {ν_s_(M–O), E_g_ symmetry}. The frequency (wavenumber) of this band slightly changes during the insertion of Cu^2+^ ion into the crystal structure as the consequence of the previously mentioned difference in the size of the metal ions. Similar spectral changes can be observed in the case of the antisymmetric bending {δ_as_(M–O), A_1g_ symmetry} at 500 cm^−1^, and the symmetric bendings {δ_s_(M–O), E_g_ symmetry} at 410 and 295 cm^−1^ for metal ions with an octahedral coordination sphere [48]. However, the position of the band at 225 cm^−1^ does not change during the Cu^2+^ insertion, rather, its intensity decreases, then totally disappears up to NP-4 (x = 0.6), similar to several peaks in the XRD diffractograms (Figure 2). This antisymmetric bending belongs to the Fe^III^–O bonds in the partly separated hematite fraction. The further peaks under 200 cm^−1^ are the signals of non-assigned external vibration modes. The intensities of these bands growth strongly together with the Cu^2+^ ratio.

**Figure 4:**
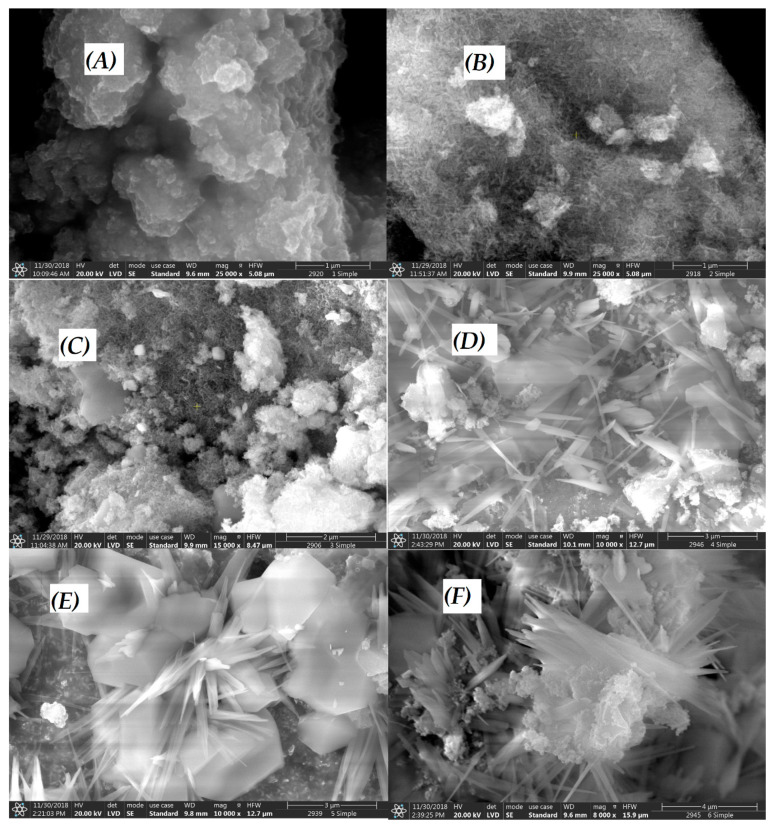
Scanning electron microscopy (SEM) images of Cu^II^_(x)_Fe^II^_(1-x)_Fe^III^_2_O_4_: (**A**) x = 0 NP-1, (**B**) x = 0.2 NP-2, (**C**) x = 0.4 NP-3, (**D**) x = 0.6 NP-4, (**E**) x = 0.8 NP-5, (**F**) x = 1 NP-6 ferrites.

SEM images of the synthesized Cu^II^_(x)_Fe^II^_(1-x)_Fe^III^_2_O_4_ NPs at various concentrations of metal salts (where x = 0.0, 0.2, 0.4, 0.6, 0.8, 1.0) are shown in Figure 4A–F. Figure 4A revealed about NP-1 (x = 0) small agglomerated nanostructures, which were totally different from the others in the series of the six NPs prepared. As a consequence of increasing Cu^2+^ ratio (x), the structure of NPs significantly changed from spherical to needle-like, embedded into clusters, in the case of NP-2 (x = 0.2, Figure 4B) and NP-3 (x = 0.4, Figure 4C). NP-4 (x = 0.6, Figure 4D) formed larger needles on the surface, while NP-5 (x = 0.8, Figure 4E) and NP-6 (x = 1, Figure 4F) in their deeper, hexagonal crystals originating from a secondary nucleation.

**Figure 5:**
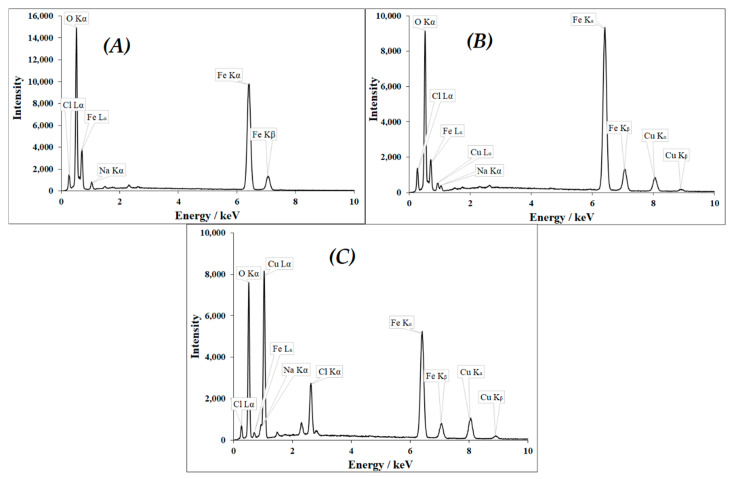
EDX spectra (recorded in scan mode) of doped ferrites (**A**) x = 0 NP-1 (**B**) x = 0.4 NP-3 and (**C**) x = 0.8 NP-5.Additionally, we have made a comparison of the EDX spectral results in spot mode, regarding the NP-5 catalyst, the SEM image of which displayed distinct needle-like and cubic crystals (see Figure 4E). The EDX spectral results for the spot containing the cubic structure (Figure S4A) displayed more intense peaks characteristic of Cl, especially at about 2.6 keV, while for that containing mostly needle-like structure (Figure S4B) more intense peaks characteristic of Fe (see at 6.3 keV) and Cu (see at 8 keV) are shown. Compared these EDX spectra to that regarding NP-5 but taken in scan mode (Figure 5C), it is clearly seen that the latter is a mixture of the previous two, indicating that this catalyst, in accordance with its SEM image (Figure 4E), involves both structures.

The EDX spectral analysis of Cu^II^_(x)_Fe^II^_(1-x)_Fe^III^_2_O_4_ NPs was carried out along with SEM in scan mode giving average intensity values for the constituents. NP-1 (x = 0, Figure 5A) showed the major characteristic peaks of Fe K_α_, Fe K_β_, Fe L_α_ and O, while smaller peaks of Na and Cl were also observed. NP-3 (x = 0.4, Figure 5B) displayed the same characteristic peaks as well as the characteristic bands of Cu: K_α_, K_β_, and L_α_. More significant Na and Cl peaks were observed in the EDX spectrum of NP-5 (x = 0.8, Figure 5C), the SEM image of which also revealed the presence of NaCl cubic crystals. The dominant impurities in the NPs were Na and Cl, originating from FeCl_3_ and NaOH applied for all precipitation reactions, and some traces of sulfur (SO_4_^2−^ anion of other metal salts), aluminum, silicon, and manganese (accompanying metal ions of iron salts) were also observed.

Since the activity of a heterogeneous (solid-phase) catalyst is frequently related to its specific surface area, this property of the prepared iron(II) doped copper ferrites was also determined by the BET method from N_2_ adsorption/desorption isotherms. As the results indicate (see in Table S3), the specific surface areas of these catalysts are in a considerable correlation with their morphology (shown in Figure 4). The catalysts consisting of mostly spherical and small needle-like structures (as NP1, NP-2, and NP-3) have significantly lower surface areas than those characterized by larger needles (NP-4, NP-5, and NP-6).

**Figure 6:**
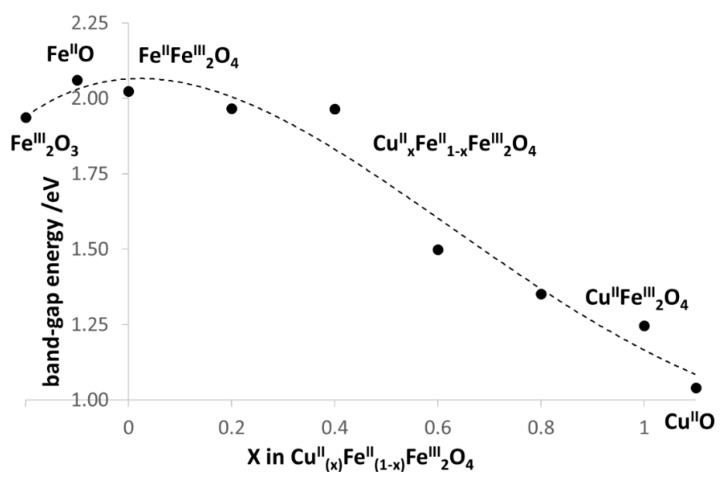
Band-gap energies (E_bg_) of doped NPs as the function of Cu^2+^content for comparison to those of the simple metal oxides.

The whole series of six NPs was analyzed for the band-gap energy (E_bg_)_._by utilization of the Kubelka-Munk function derived from the DRS spectrum. Figure S2 presents an example for the determination of E_bg_ of NP-3. As shown in Figure 6, an increase in the Cu^2+^: Fe^2+^ ratio resulted in lower band-gap energies. NP-1 (x = 0) showed higher E_bg_ of 2.02 eV (613 nm), while NP-6 (x = 1) lower E_bg_ of 1.25 eV (995 nm). It means that copper ferrites may be able to harvest the energy of near infrared light in a photocatalytic system, too. The E_bg_ values of the simple metal oxides are in good accordance with those of the doped samples. Comparing our values to those published earlier, in the cases of both simple oxides (such as Fe_2_O_3_, 2.0 eV [49] and CuO, 1.2 eV [50] and copper ferrites (2.12 to 1.90 eV for 0 to 8% Cu content [48], the corresponding band-gap energies were also in agreement.

### 3.2. Evaluation of Photocatalytic Activity of Cu^II^_(x)_Fe^II^_(1-x)_Fe^III^_2_O_4_ NPs

First of all, the potential self-degradation of MB with and without light (in dark) was checked (Table 2). In accordance with our results, earlier observations in the literature [43,44] also confirmed that MB is stable in the dark, but photosensitive in visible light. The reaction rate for this photo-induced self-degradation of MB was determined in our work, compared to that of the photocatalytic reaction, in which H_2_O_2_ was used as an oxidant with the concentration of 0.01 M suggested in similar experiments published in the literature [35]. The self-degradation of MB was ignored in subsequent studies. The presence of NPs significantly improved the relative efficiency of MB degradation (Table 2), by 34% as compared to (MB + H_2_O_2_ + Light).

**Figure 7:**
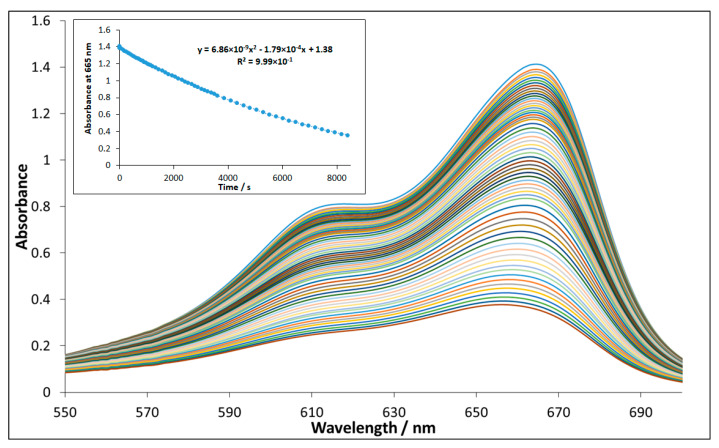
Spectral change during Methylene Blue degradation in photocatalytic system containing NP-3 (x = 0.4). The inset shows the absorbance vs. time plot at 665 nm. Concentrations: MB = 1.5 × 10^−5^ mol/L, NP-3 = 22.73 mg/L, initial pH = 7.5, and H_2_O_2_ = 1.01 × 10^−2^ mol/L.

**Figure 8:**
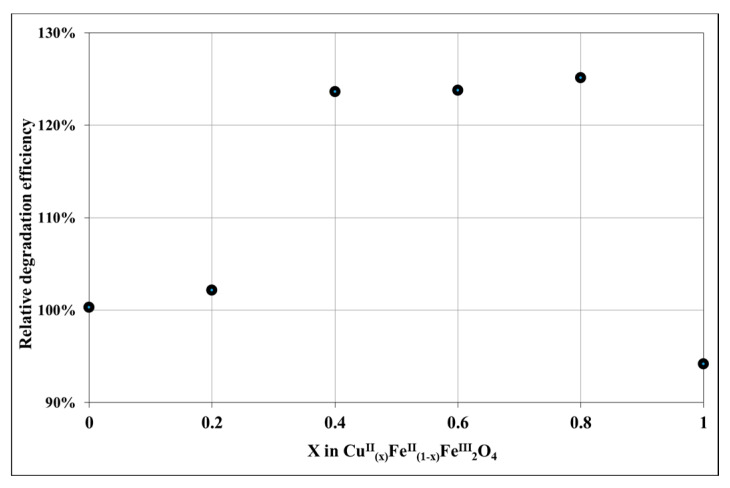
Relative photocatalytic efficiency (compared to the photodegradation of MB without catalysts) depending on the Cu^2+^:Fe^2+^ ratio in Cu^II^_(x)_Fe^II^_(1-x)_Fe^III^_2_O_4_. Concentrations were suggested by Singh et al. [35]: MB = 1.5 × 10^−5^ mol/L, NPs = 22.73 mg/L, initial pH = 7.5, and H_2_O_2_ = 1.01 × 10^−2^ mol/L.

Figure 7 shows the spectra change during the irradiation for the system containing NP-3 (x = 0.4). The decay at 665 nm (inset of Figure 7) suggests a pseudo-first-order kinetics. The logarithmic version of this plot (Figure S5) seems to confirm this expectation. However, its slight deviation from the linear function indicates the complex character of this heterogeneous process. Hence, the initial rates were used for determination of the relative efficiencies. Figure 8 reveals that in the range x = 0.2–0.8 the doped ferrite NPs showed higher degradation efficiencies as compared to the control experiment; while x = 0 (NP-1 magnetite), and x = 1 (NP-6 undoped copper ferrite) showed no remarkable change with respect to the control. The same trend was also reported in the literature for nickel doped cobalt ferrites NPs [35].

This phenomenon may originate from the fruitful combination of the structures and catalytic features of the two separated metal ferrites at given ratios. The increase of Cu^2+^ and decrease of Fe^2+^ concentrations was observed to be useful to achieve higher photocatalytic performance. SEM images revealed that x = 0.2 (NP-2) and x = 0.4 (NP-3) had small needle-like crystals. A special crystalline structure may be a determining factors of higher catalytic efficiency. Based upon this first experimental series and SEM-EDS analysis, NP-3 was selected for the further investigation of three important parameters of our heterogeneous Fenton system. Notably, the specific surface area of NP-3 is significantly lower than those of the catalysts consisting of larger needle-like crystals, indicating that this property is not crucial in the respect of their activity. Such an observation is not unusual regarding heterogeneous photocatalyst, in the case of which other (e.g., electronic or special morphologic) features are more determining.

The possible mechanism for the formation of the reactive oxygen species in the presence of Cu^II^_(x)_Fe^II^_(1-x)_Fe^III^_2_O_4_ NPs can be expressed as given below (Equations (5)–(10)). When a photon having energy *(hv)* equal to or greater than the band gap of the semiconductor photocatalyst is absorbed, an electron is promoted from the filled valence band of the semiconductor material into the vacant conduction band, creating a hole (h^+^) in the valence band. This production of electron hole pair (e‾-h^+^) promotes further reactions in the photocatalytic system.

Fe(II) + H_2_O_2_ → Fe(III) + ^●^OH + OH^−^ (5)

Fe(III) + H_2_O_2_ → Fe(II) + ^●^OOH + H^+^ (6)

H_2_O_2_ + e⁻ → ^●^OH + OH (7)

H_2_O + h^+^ → H^+^ + ^●^OH. (8)

Ionization of water under photocatalytic system:

H_2_O → OH⁻ + H^+^ (9)

OH^−^ + h^+^ → ^●^OH. (10)

**Figure 9:**
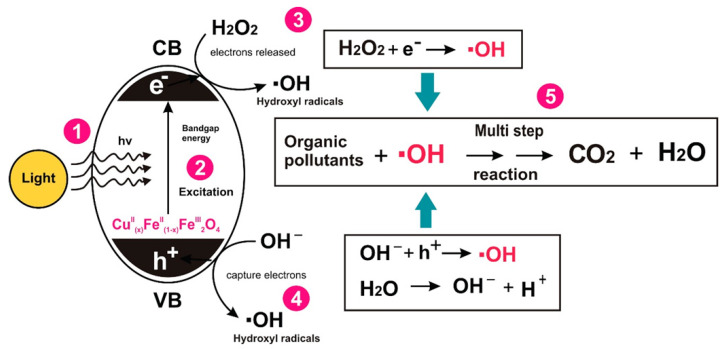
Schematic diagram for the degradation mechanism of organic pollutants in heterogeneous photo-Fenton system.

The possible mechanism of the degradation of organic pollutants in Cu^II^_(x)_Fe^II^_(1-x)_Fe^III^_2_O_4_ based photocatalysis is shown in Figure 9.

#### 3.2.1. Effect of Cu^II^_0.4_Fe^II^_0.6_Fe^III^_2_O_4_ Dosage

**Figure 10:**
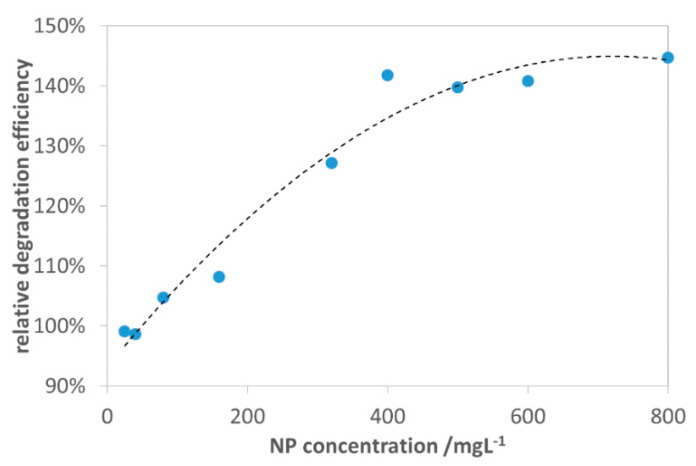
Effect of NP-3 (x = 0.4) concentration on the relative efficiency of degradation (compared to the photodegradation of MB without catalysts). Concentrations: MB = 1.5 × 10^−5^ mol/L, conc. of H_2_O_2_ = 1.01 × 10^−2^ mol/L, and initial pH = 7.5.

Based on the first photocatalytic experimental series, NP-3 (Cu^II^_0.4_Fe^II^_0.6_Fe^III^_2_O_4_) was selected for further investigation, regarding the effect of NPs dosage on the efficiency of the heterogeneous Fenton system. The NPs dosage was varied in the range of 0–800 mg/L as shown in Figure 10. It was observed that the increase in the NP-3 dosage from 0–400 mg/L showed a significant improvement in the relative efficiency of degradation by the system. This enhancement can be attributed to the higher number of active sites available for heterogeneous Fenton reactions and more photons absorbed by the catalyst particles [51]. Above 400 mg/L NPs concentration, the relative efficiency of degradation leveled off, due to the limited generation of hydroxyl radicals as a consequence of the increased turbidity of the reaction mixture, which could obstruct visible light irradiation [52]. Hence, the optimum dosage of 400 mg/L NPs was used for the further photocatalytic experiments [51].

#### 3.2.2. Effect of H_2_O_2_ Concentration

**Figure 11:**
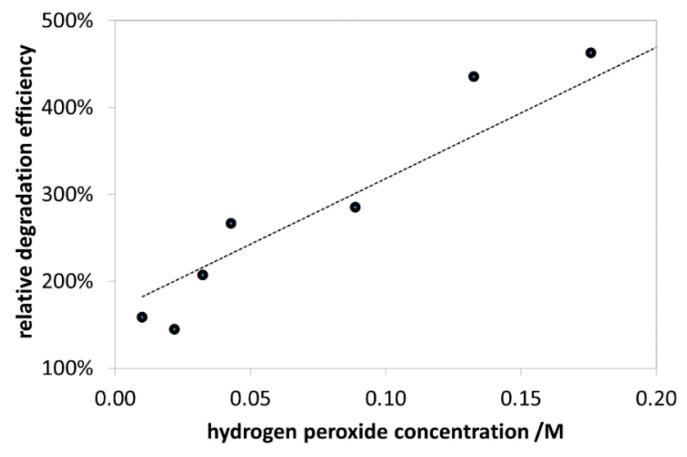
Effect of H_2_O_2_ concentration on the relative efficiency of the photocatalytic MB degradation. Concentrations: NP-3 = 400 mg/L, MB = 1.5 × 10^−5^ mol/L, and initial pH = 7.5.

The effect of H_2_O_2_ on the photocatalytic degradation of MB without NPs is shown in Figure S6. The values of this initial rate vs. hydrogen peroxide concentration plot were taken as references for the determination of the relative degradation efficiency in the presence of catalysts. As shown in Figure 11, the relative efficiency of degradation gradually increased upon enhancing the H_2_O_2_ concentration in the range of 0.01–0.18 M. It can be attributed to the production of higher amount of hydroxyl radicals at higher concentration of H_2_O_2_. The value of 0.2 mol/L used to be the upper limit for the concentration of H_2_O_2_ in the industrial applications because further increase resulted in scavenging effect on hydroxyl radicals [53]. Notably, our experiments in an extended concentration range indicated a maximum efficiency at 0.26 M (not shown). Nevertheless, 0.176 M H_2_O_2_ was used for the further experiments, not to exceed the 0.2 M limit.

#### 3.2.3. Effect of pH

**Figure 12:**
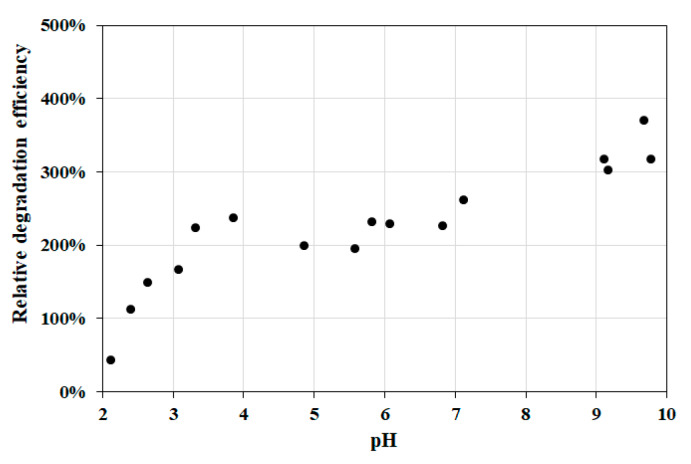
Effect of pH on the relative degradation efficiency of MB degradation. Concentrations: NP-3 = 400 mg/L, conc. of MB = 1.5 × 10^−5^ mol/L, and conc. of H_2_O_2_ = 1.76 × 10^−1^ mol/L.

Interestingly, this heterogeneous photo-Fenton system was found to be more efficient at neutral and alkaline pH as compared to conventional Fenton systems, which used to work better at lower pH. The relative degradation efficiency of extensively studied experiments was summarized in Figure 12. At pH < 3, the protonation of MB may cause the decrease of the reaction rate. Above pH 9, deprotonation of hydrogen peroxide takes place (pK_a_ = 11.75), resulting in the formation of the more reactive HO_2_^–^ species. At much higher pHs, neither the irradiation (photo-Fenton), nor the presence of metal ions (Fenton) are necessary for the effective formation of OH radicals.

#### 3.2.4. Reusability of NP-3 (Cu^II^_0.4_Fe^II^_0.6_Fe^III^_2_O_4_)

**Figure 13:**
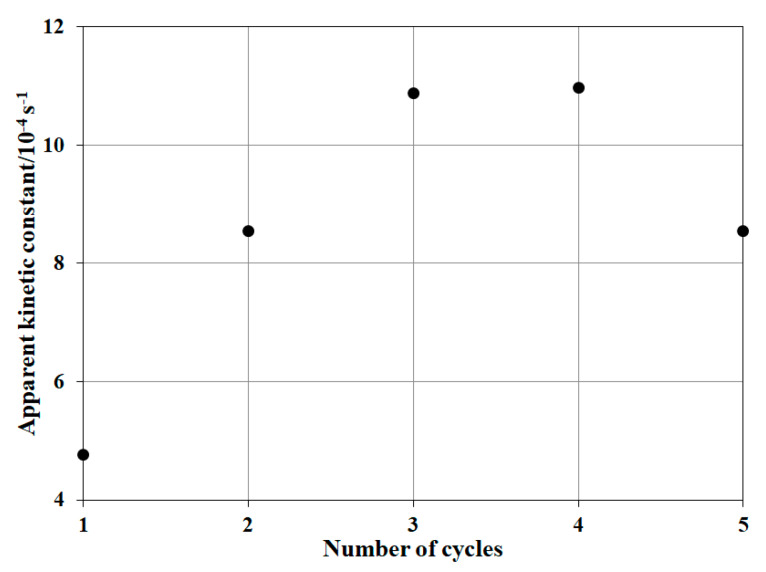
The effect of the reuse of the NP-3 catalyst on the relative efficiency the MB degradation. Concentrations: NP-3 = 400 mg/L, conc. of MB = 1.5 × 10^−5^ mol/L, pH = 7.5, and conc. of H_2_O_2_ = 1.76 × 10^−1^ mol/L.

It was observed (Figure 13) that the relative degradation efficiency increased until the third cycle. 

Its value did not change in the fourth cycle, while indicated some decrease in the fifth one. Most of the researchers reported a small decrease in the reaction rate after each cycle, but this heterogeneous Fenton system behaved quite differently, with a significant increase of the efficiency up to the fourth cycle. This phenomenon suggests that the use of the catalyst increases the accessibility of the active sites on the particle surface.

#### 3.2.5. Summarizing the Optimized Photocatalytic Conditions

**Figure 14:**
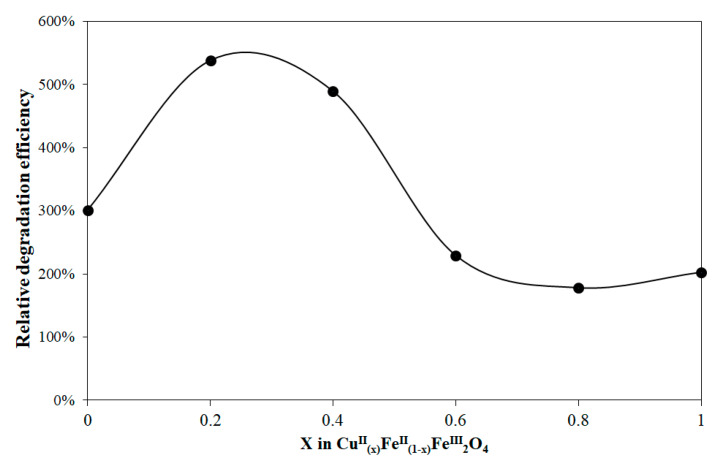
Relative photocatalytic efficiency depending on the ratio Cu^2+^:Fe^2+^ in Cu^II^_(x)_Fe^II^_(1-x)_Fe^III^_2_O_4_ at the optimized concentrations: MB = 1.5 × 10^−5^ mol/L, NPs = 400 mg/L, initial pH = 7.5, and H_2_O_2_ = 1.76 × 10^−1^ mol/L.

For the initial conditions for our experiments (Figure 7 and Figure 8), we used the already published data in the literature, suggested by Singh et al. [35]. Then the potential effects of the reactants’ concentration were revealed on the reaction rate of the photocatalytic degradation (Figure 10, Figure 11 and Figure 12). These optimized conditions differed from the initially used ones, therefore the quality of our NPs, that is, the Cu^2+^ ratio in Cu^II^_x_Fe^II^_1-x_Fe^III^_2_O_4_ was investigated again at these optimized concentrations (Figure 14). The efficiencies in Figure 14 are much higher than those in Figure 8. Hence, our whole series of doped NPs are active photocatalysts for MB degradation and our optimized concentrations are more effective. At these new conditions, NP-2 (x = 0.2) and NP-3 (x = 0.4) proved to be the best photocatalysts (Figure 14).

Although, according to the literature, 400 °C was used as the calcination temperature in the syntheses of the catalysts, after determination of the optimized application conditions for NP-3, a preliminary series of experiments regarding the effect of the calcination temperature on the activity of NP-3 was also carried out. The results indicated that above 250 °C the activity just slightly depends on the calcination temperature, and the maximum is about 300 °C (Figure S7).

## 4. Conclusions

Cu^II^_(x)_Fe^II^_(1-x)_Fe^III^_2_O_4_ nanoparticles as novel heterogeneous Fenton catalysts prepared in this work showed significant activities in the photodegradation of Methylene Blue dye. The increasing ratio of Cu^2+^ (x) in the iron(II) ferrites resulted in the decrease of the band-gap energy and the crystal size. Cu^II^_0.4_Fe^II^_0.6_Fe^III^_2_O_4_ (NP-3) proved to be the most active photocatalyst among the series of six NPs, partly due to its transition structure containing both spherical and small needle-like particles. At the optimized conditions, the efficiency for MB degradation was 6 times higher in the presence of photocatalysts than that in its absence. Contrary to other heterogeneous Fenton systems, our catalysts exhibit a higher efficiency at neutral and alkaline pH, as well as better reusability. Our results unambiguously indicate that this type of NP can be used in heterogeneous Fenton systems to efficiently remove toxic organic compounds from wastewaters.

## Figures and Tables

**Table 1:** Design of experiments for nanoparticles (NPs) synthesis.

Cu^II^_(x)_Fe^II^_(1-x)_Fe^III^_2_O_4_	x = 0	x = 0.2	x = 0.4	x = 0.6	x = 0.8	x = 1
Sample name	NP-1	NP-2	NP-3	NP-4	NP-5	NP-6
Fe(NH_4_)_2_(SO_4_)_2_∙6H_2_O (g)	1.961	1.569	1.176	0.784	0.392	0.000
FeCl_3_∙6H_2_O (g)	2.703	2.703	2.703	2.703	2.703	2.703
CuSO_4_ (g)	0.000	0.160	0.319	0.479	0.638	0.798

**Table 2:** Control experiments for MB degradation. Concentrations: MB = 1.5 × 10^−5^ mol/L, NP-3 = 22.73 mg/L, H_2_O_2_ = 1.01 × 10^−2^ mol/L.

Experiment	Reaction Rate (M/s)	Relative Efficiency of Degradation
MB + NPs + Light	1.24 × 10^−10^	41.8%
MB + Light	1.13 × 10^−10^	38.3%
MB + H_2_O_2_	2.58 × 10^−11^	8.7%
**MB + H_2_O_2_ + Light**	**2.95 × 10^−10^**	**100.0%** **(the basis of comparison)**
MB + NPs + H_2_O_2_ + Light	3.96 × 10^−10^	133.9%

## Supplementary Materials

The following are available online at https://www.mdpi.com/2079-4991/10/5/921/s1, Figure S1: Chemical structure of Methylene Blue; Figure S2: Kulbelka-Munk function for determination the band-gap energy (E_bg_) of NP-3; Figure S3: Particle size distribution of NP-3; Figure S4: EDX spectra (recorded in spot mode) of the NP-5 catalyst (Cu^II^_(x)_Fe^II^_(1-x)_Fe^III^_2_O_4_, x = 0.8), regarding the spot on cubic (A) and needle-like (B) structure; Figure S5: The logarithm of the absorbance at 665 nm vs. time plot for the degradation of MB (see the inset of Figure 7). Concentrations: MB = 1.5 × 10^−5^ mol/L, NP-3 = 22.73 mg/L, initial pH = 7.5, and H_2_O_2_ = 1.01 × 10^−2^ mol/L.; Figure S6: Effect of H_2_O_2_ concentration on MB degradation in the absence of NP. Concentrations: MB = 1.5 × 10^−5^ mol/L, and initial pH = 7.5. (The kinetic constants were determined from the initial rates.); Figure S7: Effect of the calcination temperature of the NP-3 (x = 0.4) catalyst on the apparent kinetic constant of MB degradation. Concentrations: NP-3 = 400 mg/L, MB = 1.5 × 10^−5^ mol/L, conc. of H_2_O_2_ = 1.76 × 10^−1^ mol/L, and initial pH = 7.5. Text S1: Precipitation of metal hydroxides; Table S1: Theoretical and experimental Cu/Fe ratios of the catalysts prepared; Table S2: Specific surface areas (BET) of the catalysts prepared.
