# Supplementary material for: Synthesis, Characterization and Application of Iron(II) Doped Copper Ferrites (CuII(x)FeII(1-x)FeIII2O4) as Novel Heterogeneous Photo-Fenton Catalysts
Source: Nanomaterials (Basel). 2020 May 9;10(5):921. doi: 10.3390/nano10050921 (PMC7279361; doi:10.3390/nano10050921)
Supplement: Supplementary file 1 [file nanomaterials-10-00921-s001.pdf]

Supplementary Materials

Synthesis, Characterization and Application of Iron(II) Doped Copper Ferrites ($Cu^{II}_{(x)}Fe^{II}_{(1-x)}Fe^{III}_2O_4$) as Novel Heterogeneous Photo-Fenton Catalysts

Asfandyar Khan [1,2], Zsolt Valicsek [1] and Ottó Horváth [1,*]

1 Department of General and Inorganic Chemistry, Faculty of Engineering, University of Pannonia, Egyetem utca 10, H-8200 Veszprém, Hungary; asfandyarkhan100@gmail.com (A.K.); valicsek@almos.uni-pannon.hu (Z.V.)
2 Department of Textile Processing, National Textile University, 37610 Faisalabad, Pakistan
* Correspondence: horvath.otto@mk.uni-pannon.hu

Received: 31 March 2020; Accepted: 6 May 2020; Published: date

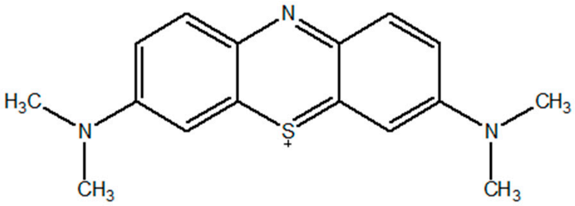

Figure S1. Chemical structure of Methylene Blue.

Text S1. Precipitation of metal hydroxides

The values given in the manuscript for the Cu/Fe ratio in each catalyst sample are not only theoretical values. As indicated in Table 1, the Cu/Fe ratios have been adjusted by the corresponding masses of the metal salts weighed into the reaction mixture of synthesis. Since 5M NaOH was applied in excess for the precipitation, the total amounts of the metal ions weighed in were precipitated, due to the very low values of the solubility product constants (K_{sp}) [41]:

$Fe(OH)_2$ 8.00×10^{-16} M^3
$Fe(OH)_3$ 2.79×10^{-39} M^4
$Cu(OH)_2$ 2.20×10^{-20} M^3

On the basis of these K_{sp} values, the theoretical concentrations in the solution phase were

$Fe(OH)_2$ 3.20×10^{-17} M
$Fe(OH)_3$ 2.23×10^{-41} M
$Cu(OH)_2$ 8.80×10^{-22} M

Besides, no formation of hydroxo complexes occur in these systems.

Table S1. Theoretical and experimental Cu/Fe ratios of the catalysts prepared.

$Cu^{II}_{(x)}Fe^{II}_{(1-x)}Fe^{III}_2O_4$	x = 0.2	x = 0.4	x = 0.6	x = 0.8	x = 1
Sample name	NP-2	NP-3	NP-4	NP-5	NP-6
Theoretical Cu/Fe ratio	0.071	0.154	0.250	0.364	0.500
Experimental Cu/Fe ratio*	0.068	0.148	0.244	0.353	0.479
Deviation (%)	4.22	3.90	2.40	3.02	4.20

*Determined by ICP measurements.

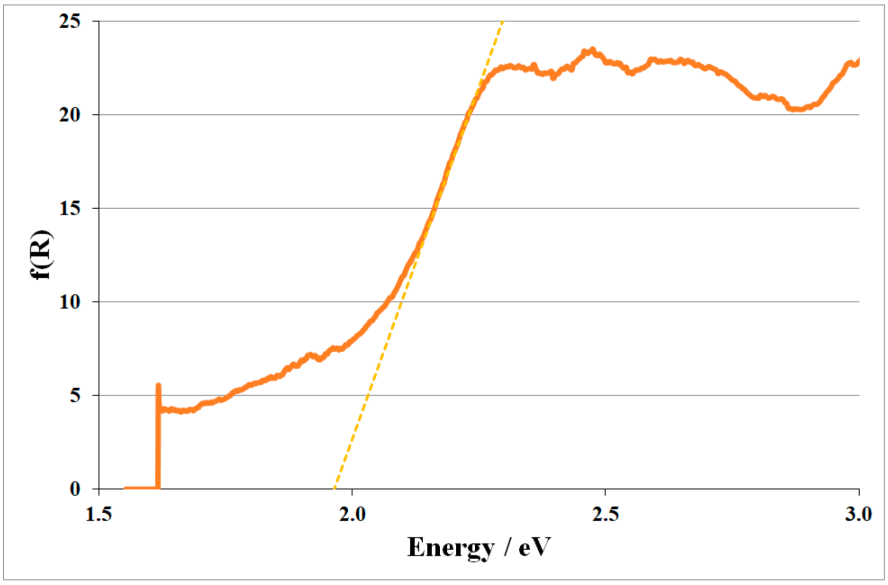

Figure S2. Kulbelka-Munk function for determination the band-gap energy (E_{bg}) of NP-3.

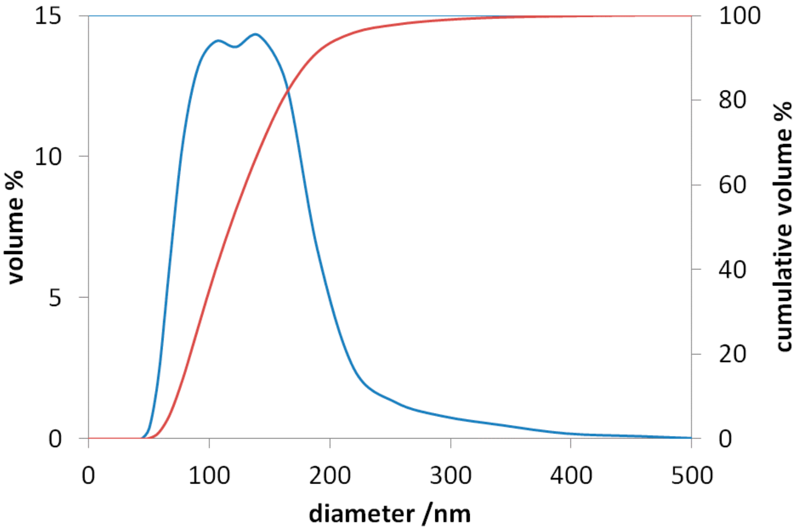

Figure S3. Particle size distribution of NP-3.

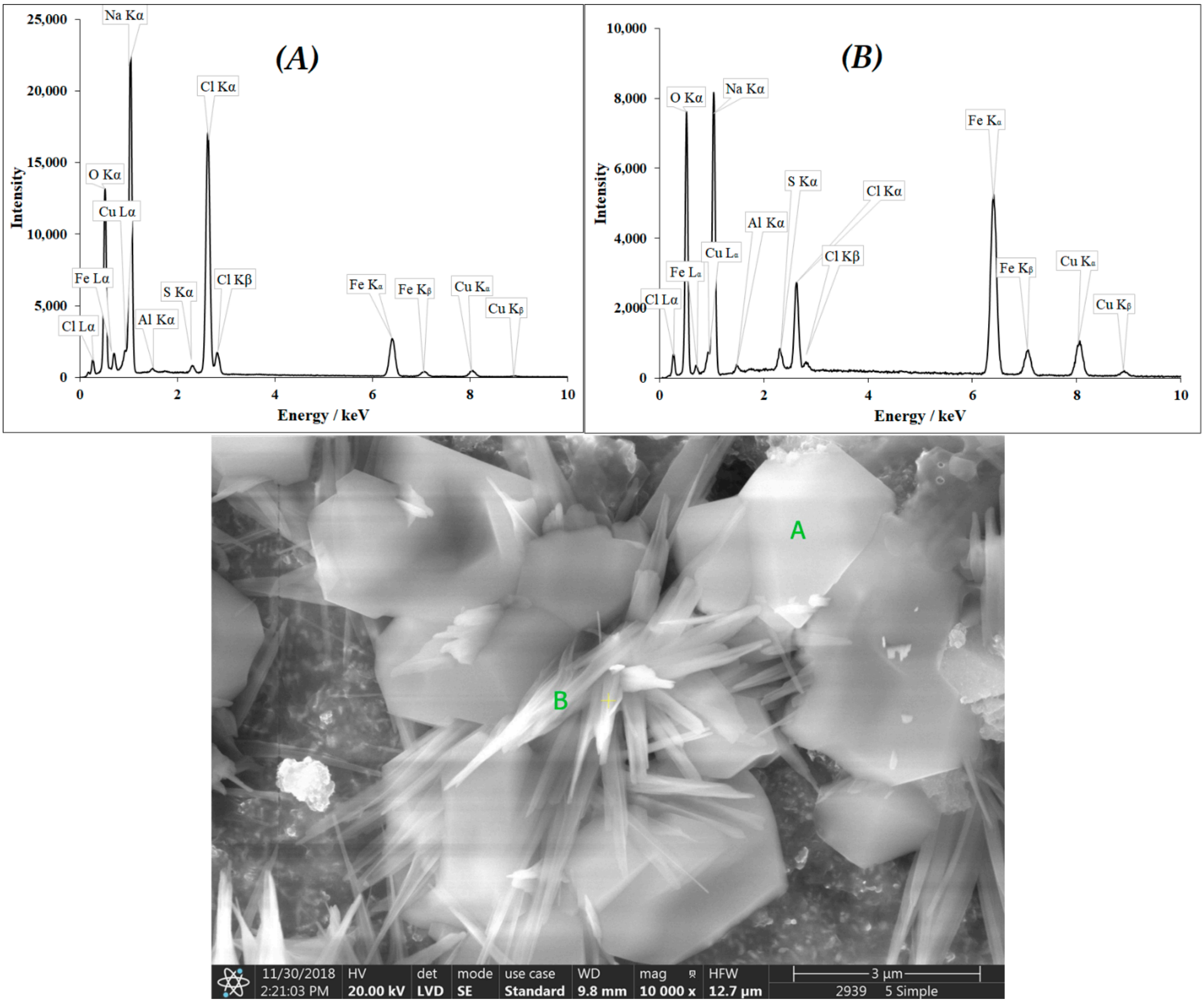

Figure S4. EDX spectra (recorded in spot mode) of the NP-5 catalyst ($Cu^{II}_{(x)}Fe^{II}_{(1-x)}Fe^{III}_2O_4$, x=0.8), regarding the spot on cubic (**A**) and needle-like (**B**) structure.

Table S2. Specific surface areas (BET) of the catalysts prepared.

$Cu^{II}_{(x)}Fe^{II}_{(1-x)}Fe^{III}_2O_4$	x = 0	x = 0.2	x = 0.4	x = 0.6	x = 0.8	x = 1
Sample name	NP-1	NP-2	NP-3	NP-4	NP-5	NP-6
Specific surface area (BET) / $m^2\ g^{-1}$	11.1	20.8	26.0	62.7	64.1	59.3

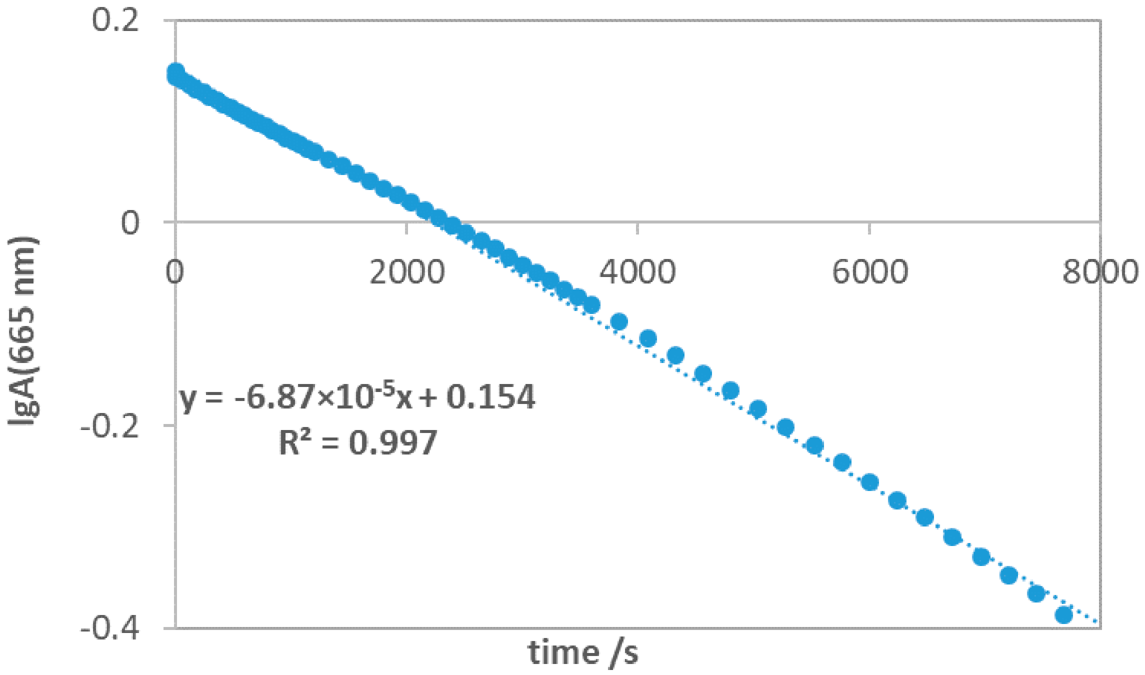

Figure S5. The logarithm of the absorbance at 665 nm vs. time plot for the degradation of MB (see the inset of Fig. 7). Concentrations: MB = 1.5 × 10^{-5} mol/L, NP-3 = 22.73 mg/L, initial pH = 7.5, and H_2O_2 = 1.01 × 10^{-2} mol/L.

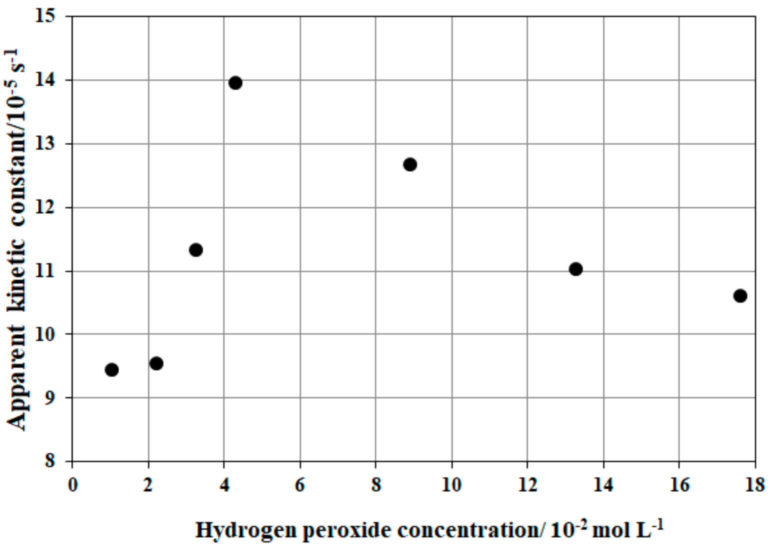

Figure S6. Effect of H_2O_2 concentration on MB degradation in the absence of NP. Concentrations: MB = 1.5 × 10^{-5} mol/L, and initial pH = 7.5. (The kinetic constants were determined from the initial rates.)

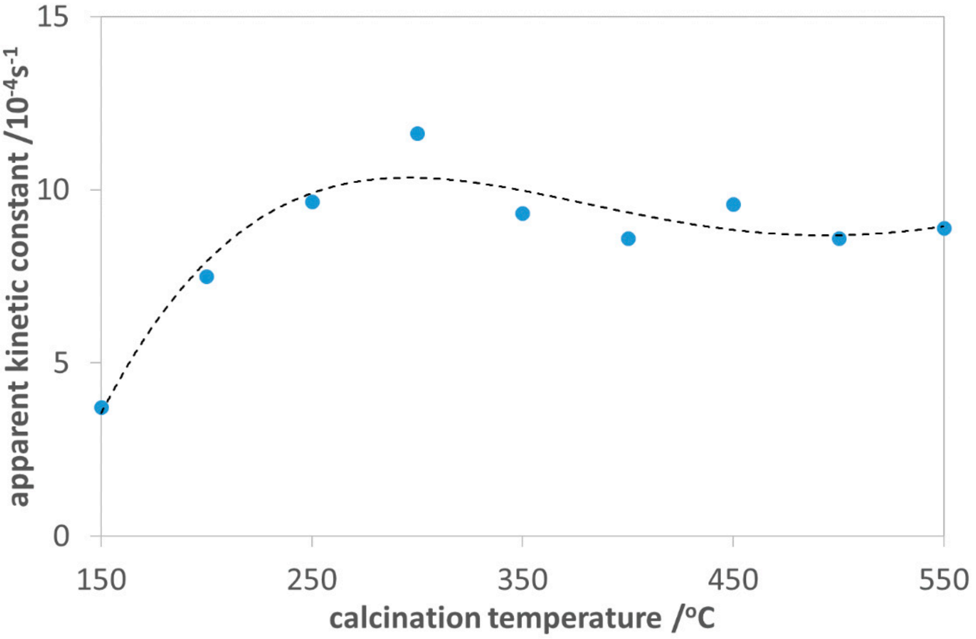

Figure S7. Effect of the calcination temperature of the NP-3 (x = 0.4) catalyst on the apparent kinetic constant of MB degradation. Concentrations: NP-3 = 400 mg/L, MB = 1.5×10^{-5} mol/L, conc. of H_2O_2 = 1.76×10^{-1} mol/L, and initial pH = 7.5.
